# Age differences in virtual environment and real world path integration

**DOI:** 10.3389/fnagi.2012.00026

**Published:** 2012-09-25

**Authors:** Diane E. Adamo, Emily M. Briceño, Joseph A. Sindone, Neil B. Alexander, Scott D. Moffat

**Affiliations:** ^1^Eugene Applebaum College of Pharmacy and Health Sciences, Wayne State UniversityDetroit, MI, USA; ^2^Institute of Gerontology, Wayne State UniversityDetroit, MI, USA; ^3^Department of Psychology, Wayne State UniversityDetroit, MI, USA; ^4^Department of Linguistics, University of MichiganAnn Arbor, MI, USA; ^5^Mobility Research Center, Geriatrics Center and Division of Geriatric Medicine, University of Michigan Hospitals and Veterans Affairs Ann Arbor Health Care System Geriatric Research Education and Clinical CenterAnn Arbor, MI, USA; ^6^School of Psychology, Georgia Institute of TechnologyAtlanta, GA, USA

**Keywords:** aging, Alzheimer's disease, hippocampus, navigation, path integration, spatial memory

## Abstract

Accurate path integration (PI) requires the integration of visual, proprioceptive, and vestibular self-motion cues and age effects associated with alterations in processing information from these systems may contribute to declines in PI abilities. The present study investigated age-related differences in PI in conditions that varied as a function of available sources of sensory information. Twenty-two healthy, young (23.8 ± 3.0 years) and 16 older (70.1 ± 6.4 years) adults participated in distance reproduction and triangle completion tasks (TCTs) performed in a virtual environment (VE) and two “real world” conditions: guided walking and wheelchair propulsion. For walking and wheelchair propulsion conditions, participants wore a blindfold and wore noise-blocking headphones and were guided through the workspace by the experimenter. For the VE condition, participants viewed self-motion information on a computer monitor and used a joystick to navigate through the environment. For TCTs, older compared to younger individuals showed greater errors in rotation estimations performed in the wheelchair condition, and for rotation and distance estimations in the VE condition. Distance reproduction tasks (DRTs), in contrast, did not show any age effects. These findings demonstrate that age differences in PI vary as a function of the available sources of information and by the complexity of outbound pathway.

## Introduction

An important aspect of spatial navigation is path integration (PI) (Mittelstaedt and Mittelstaedt, 1982; Cheng and Spetch, 1998), which refers to the ability to monitor one's position in space using self-motion cues derived from visual (linear and radial optic flow), vestibular (translational and rotational accelerations), and/or proprioception (feedback from muscles, tendons, and joints). Optic flow refers to the processing of the flow of visual information resulting from movement of the observer. Decline in the ability to process optic flow has been linked with declines in navigation abilities observed with early onset of Alzheimer's disease (de Ipolyi et al., 2007). Vestibular cues derived from sensory receptors in the middle ear (utricle, saccule, and semicircular canals) detect differences in linear and angular accelerations. Detecting changes in the speed of translation (distance-specific) and rotation (direction-specific relative to global spatial coordinates) contribute to updating spatial information used in PI tasks. Support for the importance of vestibular information in PI is evidenced by the observation that labyrinthine-defective human subjects have difficulty estimating and reproducing the length of a path or a previously seen target (Glasauer et al., 1994, 1999). Proprioception provides information about body and limb position awareness and is used to mediate one's conscious perception of movement (Goodwin et al., 1972; McCloskey, 1978; Gandevia et al., 1992) and encode body movements in space (Chance et al., 1998; Mittelstaedt and Mittelstaedt, 2001). Thus, these primary sensory systems all contribute significantly to the ability to navigate one's environment.

Studies on ants (Cheng et al., 2009), bees (Frisch, 1967), and, particularly, rodents (Mittelstaedt and Mittelstaedt, 1982; Etienne et al., 1988) show that when eliminating visual and olfactory cues (Etienne et al., 1986) animals are able to use PI effectively (Mittelstaedt and Mittelstaedt, 1982; Jeffery et al., 1997). Although sensory-specific contributions to PI vary within species, bees rely on optic flow (Srinivasan et al., 2000) and ants determine distance through proprioceptors (Wohlgemuth et al., 2001). In rats, recordings from anterior thalamic head-direction cells show that vestibular and visual movement cues, along with motor efferent information (Zugaro et al., 2001) work interactively to determine the directional frame of reference during PI tasks (Sharp et al., 1995; Blair and Sharp, 1996).

Although specific procedures employed to evaluate *human* PI vary, paradigms typically restrict participants to a subset of self-motion cues (visual, vestibular, and proprioceptive) in order to evaluate the relative contribution of sensory input (Loomis et al., 1993; Chance et al., 1998; Philbeck et al., 2008). To restrict individuals to proprioceptive and vestibular information, participants typically wear a blindfold and noise-blocking headphones and are then required to reproduce movements through which they were transported. Individuals may be moved along a single linear distance and be asked to return to the origin by reproducing the distance they traveled by moving in the opposite direction. In contrast, triangle completion tasks (TCTs) are more complicated in that participants may be moved along two legs of a triangle and then when reaching the end of the second leg of the triangle, must rotate and return to the origin without assistance. In this task, spatial components associated with angles turned and distances traveled must be successfully integrated in order to approximate the return to the origin.

Healthy young adults are fairly accurate when reproducing distances and angular rotations (Mittelstaedt and Mittelstaedt, 1982, 2001; Israel and Berthoz, 1989; Glasauer et al., 1994; Israel et al., 1995; Ivanenko et al., 1997; Marlinsky, 1999). In addition, Wiener et al. (2011) showed that head orientation assists with deriving a homing vector for locomotion tasks performed in the absence of visual and auditory information. However, when required to combine linear and rotational displacements simultaneously (as in TCTs), healthy young adults demonstrate greater difficulty with the task, with errors mainly driven by the rotation component of the movement (Loomis et al., 1993; Marlinsky, 1999). Further, the contribution of motor-efferent signals derived from self-movements cannot be ignored as a source of information contributing to PI abilities (Mittelstaedt and Mittelstaedt, 2001).

Despite numerous demonstrations of age-related declines in navigation in both human (Moffat and Resnick, 2002; Driscoll et al., 2005; Cushman et al., 2008; Rodgers et al., 2012) and non-human (Ingram, 1988; McLay et al., 1999) species, few studies have investigated possible age differences in PI. Allen et al. (2004) found that older adults performed similarly to young adults when returning to the origin of a triangle under guided walking conditions. However, when performing this same task following passive conveyance in a wheelchair, older individuals showed greater errors than young individuals, suggesting that performance is related to the amount of sensory information available. That is, age differences are minimized when both proprioceptive and vestibular perceptual signals are available. In a study designed to examine age-differences in visual PI, Mahmood et al. (2009) found that older compared to younger adults had greater difficulty in a TCT presented on a computer. Similar age effects in PI were observed by Harris and Wolbers (2012) using a virtual environment (VE).

Although age-related declines in PI have been demonstrated in one real-world and two VE studies, the extent to which each of the sources of sensory information contribute to these differences is not known as no study has investigated all three sensory systems in the same participants. This is critical as each sensory system known to contribute to PI, shows some age-related decline (Adamo et al., 2007, 2009; Horning and Gorman, 2007; Kulmala et al., 2008). Understanding the contribution of sensory-specific information to PI abilities may contribute to improving interventions to offset age-associated declines in wayfinding abilities that rely on the integration of sensory specific information (Loomis et al., 1993; Chance et al., 1998; Philbeck et al., 2008) and cognitive functions (Davis et al., 2008). Therefore, the present study investigated the relative contribution of three sources of sensory information to age-related differences in PI. The contribution of various sources of sensory information to PI was investigated by providing conditions that reduced a source of sensory information. For blindfolded/auditory blocking walking conditions self-motion cues were derived primarily from proprioceptive and vestibular cues. Motor efferent information is also available. For blindfolded/auditory blocking wheelchair propulsion conditions self-motion cues were primarily derived from vestibular cues since they were seated in a wheelchair and only performed active movements when operating the joystick during return paths. During VE conditions, self-motion cues were derived from optic flow and there were no visual landmark or cues available. Passive viewing was completed before individuals used a joystick to complete return paths. For outbound paths, sensory information was specific to each condition. In the wheelchair and VE conditions return paths required the use of a joystick to complete task requirements. The focus of this study investigated PI abilities derived specifically from self-movement cues.

## Methods

Thirty-eight, right handed, young (*n* = 22, 10 female, mean age = 23.8 ± 3.0 years) and older (*n* = 16, 11 female, mean age = 70.1 ± 6.4 years) adults participated in this study. Participants were free from any neurological or musculo-skeletal conditions that may have impaired performance and scored >28/30 on the Mini Mental State Exam (MMSE) (Folstein et al., 1975). Visual acuity was 20/40 or higher for all individuals. Participants were recruited from the Metropolitan Detroit/Ann Arbor area and Wayne State University. Informed consent approved by the Institutional Review Board at the University of Michigan and the Human Investigation Committee at Wayne State University was completed before each testing session.

### Screening tools and assessments

Vision and mobility assessments were administered to all participants. Tests for contrast sensitivity (Mars Letter Contrast Sensitivity Test) and color vision (Ishihara Color Plates Test) ensured that any potential visual deficits would not interfere with their ability to perform the VE condition. The Timed Up and Go task (Podsiadlo and Richardson, 1991) assessed safe walking ability. To determine whether the participants experienced any symptoms of motion sickness following performance of the tasks in the VE, the 16-item Nausea Symptom Questionnaire was administered.

### Experimental set-up

Two real world conditions and one VE condition were used to assess PI abilities. The real world conditions consisted of guided walking and wheelchair propulsion and took place in a human research laboratory that was 12.2 × 9.1 m. For both real world conditions, participants wore a halo structure on their head. This consisted of a rigid plastic structure that was placed on the head in a position parallel to the ground. The halo was embedded with eight equally distributed reflective markers (Light Emitting Diodes, LEDs) that were connected with a thin cable and then to a transmitter. The transmitter powered the reflective markers, and the position of the reflective markers embedded in the halo was recorded by four Optotrak Certus™ position sensors (cameras) placed in the testing volume. The reflective markers tracked whole body movements.

For the walking condition, a gait belt worn by the participant and lightly grasped by the experimenter was used to steer the participants through the distances traveled and angles turned during the walking task. Participants also wore noise-reducing earphones and a blindfold to suppress auditory and visual information. Comfortable walking shoes were worn.

For the wheelchair condition, participants were comfortably seated in an IMC Heartway Rumba S HP with a Dynamic DL 5.2i armrest control unit mounted on the right side of the chair. Individuals moved a joystick device with their right hand to execute forward, backward, and turning movements while seated in the chair.

The VE condition was administered using a modified version of Unreal Tournament 2003 and the software package Unreal Editor 3.0 (Epic Games, Inc.). In a dimly lit room, participants were seated 23″ away from an 18″ flat screen LCD monitor. Seat height was adjusted so that the eyes were level with the midpoint of the computer monitor. A Thrustmaster Top Gun Fox 2 Pro joystick mounted on top of a 27 5/8th in. platform was placed on the right side of the individual. The VE condition consisted of passively viewing a movement trajectory on a computer monitor, then maneuvering a joystick when responding to the requirements of the active portion of the task (see below).

### Experimental procedures

Following completion of screening tools, participants performed PI tasks in the following order: walking, wheelchair propulsion, and VE. The VE was presented last because of concerns that motion sickness or dizziness induced by VE navigation may affect performance on the real world tasks and may increase risk for falls in the blindfolded walking condition. For each condition, two tasks were performed that consisted of: (1) rotating and returning to the origin of a triangle and (2) reproducing a linear distance traveled.

### Real world-triangle completion and distance reproduction tasks

**Figure 1:**
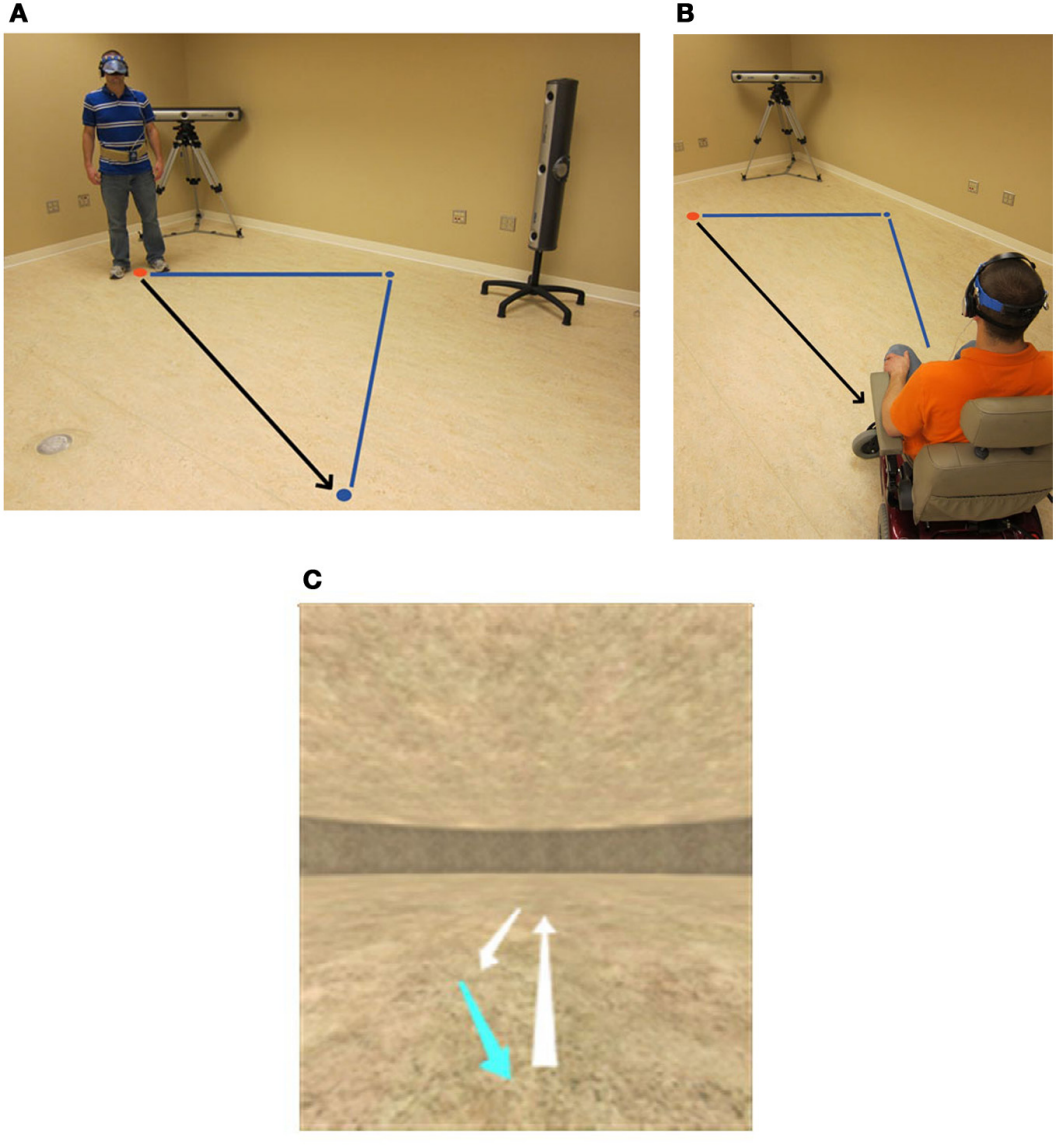
**Schematic of experimental setup: participants wore noise reducing headphones and a blindfold during guided walking (A) and wheelchair propulsion (B) conditions.** Data were collected from reflective markers placed on a halo structure worn on the head of the participant. VE condition consisted of passively viewing a movement trajectory on a computer monitor **(C)** then moving a joystick when responding to the active portion of the task. Guided movements (lighter shaded lines) and participants movements (darker shaded lines) are shown on the schematic.

The TCT involved passive motion of two linear distances separated by one angular rotation. For real-world walking, participants were guided along the first leg of a triangle and brought to a complete stop. The experimenter then rotated and guided participants through the second leg of the triangle. After stopping at the end of the second leg, participants were signaled to rotate and walk to the origin. For real world wheelchair propulsion, participants were seated in the chair and the experimenter guided the movement of the wheelchair by standing behind it and reaching forward to activate the joystick. The experimenter followed a path outlined on the floor that indicated the precise distance required to travel and angle to turn. For return paths, participants either walked (walking condition) or propelled their wheelchair (wheelchair condition) back to the origin (see Figures 1A,B, respectively). After their position was recorded, they were moved away from their end position and briefly removed the blindfold in order to allow them to re-orient to the environment. This reduced the potential desire to remove the blindfold at unscheduled moments during subsequent trials when they may perceive some confusion about their location. Triangles were counter-balanced according to the length of the leg and angle turned. The length of the first and second leg ranged from 1.2 to 5.5 m and the turning angle was either 36 or 104°. The starting position for each triangle was varied.

In the distance reproduction task (DRT), participants were guided through a linear displacement of 3.0, 6.1, and 9.1 m. When they came to a complete stop, participants were then rotated toward the origin and signaled to travel back to the starting position. After their end position was recorded, participants were escorted away from the end position before briefly removing the blindfold as indicated above. The starting position varied for each distance.

In order to ensure the experimenter provided a similar walking and wheelchair propulsion velocity for young and older individuals, a sub analysis of movements was analyzed. The walking speed for the experimenter was 0.78 m/sec and 0.69 m/sec, for young and older participants, respectively, and these differences were not statistically significant (*p* = 0.45). Likewise, the movement speed for the experimenter when pushing the wheelchair was 0.65 m/sec for younger individuals and 0.56 m/sec for older individuals. These differences were not statistically significant (*p* = 0.17). Similar velocities (0.6 m/sec) were used by the experimenter during walking and wheelchair propulsion conditions in the Allen et al. (2004) study.

### Virtual environment-triangle completion and distance reproduction tasks

When performing the TCT and DRT in the VE, participants first passively viewed motion of the linear path for the DRTs and then the outbound paths, separated by a rotation for the TCTs (see Figure 1C). Speeds of movement during passive viewing were matched for both the TCT and DRT.

Three DRTs (3.0, 6.1, and 9.1 m) and six TCTs were completed each condition (walk, wheelchair, and VE). One additional practice trial preceded two test trials for each condition × task combination (see Table 1 for details about the triangles).

**Table 1:** **Six triangles were presented to the participants**.

	**T1**	**T2**	**T3**	**T4**	**T5**	**T6**
Leg 1 (m)	1.2	5.5	2.4	1.8	2.4	5.5
Turning angle (degree)	36	36	36	104	104	104
Leg 2 (m)	1.8	2.4	5.5	1.2	5.5	2.4

The distance of the first and second leg and turning angle were counterbalanced across triangles. Triangles performed in the VE were scaled identically.

### Practice session

For real world conditions, participants were familiarized with the testing environment by performing specified movements in the testing area. For the walking condition, individuals were guided through the movement by the experimenter, first without, then while wearing the gait belt, blindfold, and noise blocking earphones. The experimenter explained to the participants that a tap on the shoulder would be used to indicate reference movement onset and movement termination. When the reference movement distance was completed, participants were rotated and then instructed to reproduce the distance traveled or to return to the origin of the triangle. Two practice trials were performed to ensure individuals understood the instructions.

For the wheelchair condition, participants were seated in the wheelchair and were requested to travel in a straight line and maneuver around cones placed equidistance apart on the floor by moving the joystick located on the armrest of the wheelchair. Following this, the experimenter propelled the participant through the testing environment. This was done first with and then without the participant wearing the blindfold and noise blocking earphones. Likewise, the experimenter explained that a tap on the shoulder would be used to indicate reference movement onset and movement termination. Additional time (approximating 15 min) was provided to practice this task when compared to that needed to practice walking tasks, in order to allow the individual to become comfortable using the controls.

A practice session was also provided prior to participating in the VE condition. Participants viewed a cubic room that contained landmarks. They first were provided with instruction on the use of the joystick and then used the joystick to move freely around the room. When participants demonstrated competence with the use of the joystick in moving freely around the room, competency with joystick control was verified by completing a speed task. The speed task consisted of traveling through a long and circuitous virtual hallway; participants were required to travel through the hallway in 120 s or less to verify joystick competence.

### Testing session

For real world conditions, the experimenter guided the participants through the testing paths, and then tapped them on the shoulder to signal when it was time to reproduce the distance traveled or return to the origin of the triangle. When individuals moved beyond the detectable range of the positions sensors, distance and angular measurements of end positions were taken with a measuring tape and goniometer. Trials were also terminated if safety was a risk (for example, if bumping into a wall was imminent). For the VE condition, participants first passively viewed motion of a linear distance in a long hallway, and then were asked to use a joystick to travel the same distance they previously viewed. Speeds of movement during passive and active viewing were deliberately mismatched to prevent participants from using a simple time-counting strategy to perform reproductions. Participants were informed of this mismatch and told explicitly not to use time estimation to reproduce the distances and that attempting to do so would be futile.

### Data collection

For real world conditions, movement data were recorded from the Optotrak Certus™ motion capture system. Movement data detected from at least three active markers on the halo were used to calculate a centroid using Matlab software that, in turn, provided a single trajectory corresponding to each person's movements. When movements were beyond the detectable range of the position sensors, floor measurements were taken. Movement data for the VE condition were collected within Unreal Tournament 2003. Data collected for all conditions were processed using customized Labview software.

### Data analysis

In order to evaluate the error across different distances, the relative error was calculated for each condition (walk, wheelchair, and VE) and task (DRT, TCT) combination.

The relative distance error for DRT and TCT was calculated as a proportion of the distance traveled relative to the required distance, (Relative distance error = |*dactual* – *d*required|/*d*required). The relative rotation error for TCT was calculated as a proportion of the angle turned relative to the required rotation, (Relative rotation error = |*ractual* – *r*required|/*r*required). Age differences for assessments and control measures were reported as means ± SD.

## Results

Independent sample *t*-tests showed no statistically significant differences for MMSE scores (29.0 ± 1.2 and 28.5 ± 1.4) years of education (15.6 ± 1.9 and 15.0 ± 2.0), motion sickness symptoms or fatigue between young and old groups (*p* > 0.05), respectively. In addition, there were no statistically significant differences between young and older individuals for visual screening and walking assessments or reports of motion sickness post-participation in the VE condition (*p*'s > 0.05) (see Table 2).

**Table 2:** **Mean (SD) for screening assessments in young and older adults**.

	**Young**	**Older**	***P*-values**
MMSE	29.0 (1.2)	28.5 (1.4)	0.13
Education (years)	15.6 (1.9)	15.0 (2.0)	0.41
Motion Sickness	2.1 (1.7)	1.9 (1.9)	0.48
Fatigue	0.27 (0.45)	0.38 (0.50)	0.51
Fatigue (% reporting 0/1)^*^	72.7/27.3	62.5/37.5	0.50
Contrast sensitivity	1.7 (0.04)	1.7 (0.06)	0.23
Ishihara color-blindness	20.2 (1.0)	20.4 (0.8)	0.58
TUG (s)	8.4 (1.7)	9.1 (2.3)	0.23
Speed test (s)	93.3 (3.2)	100.4 (5.7)	0.08

Independent t-tests were conducted with the exception of chi square test

* .

### Relative error for triangle completion tasks

Analyses of the TC tasks involved decomposing the error as a function of the required linear distance traveled and angle turned to understand how each individual component contributed to overall performance. A repeated measures ANOVA was conducted to test for main and interaction effects for condition (walk, wheelchair, and VE) and triangle (1–6) with age as the between subjects factor and relative distance error and relative rotation error as dependent variables.

There was a significant main effect for age [*F*_(1, 36)_ = 6.8, *p* < 0.05] with older adults showing greater errors than young adults and a significant main effect for condition [*F*_(2, 72)_ = 22.8, *p* < 0.0001] with greater errors found in the VE condition when compared to the wheelchair; and in the wheelchair condition when compared to the walking condition. There were no significant main effects of triangle [*F*_(5, 180)_ = 1.1. *p* > 0.05], thus triangle was collapsed across conditions for subsequent comparisons and no significant main effects for gender [*F*_(1, 36)_ = 0.22, *p* > 0.05]. A significant two-way age x condition interaction [*F*_(2, 72)_ = 7.6, *p* < 0.01] indicated that age differences were specific to condition tested.

**Figure 2:**
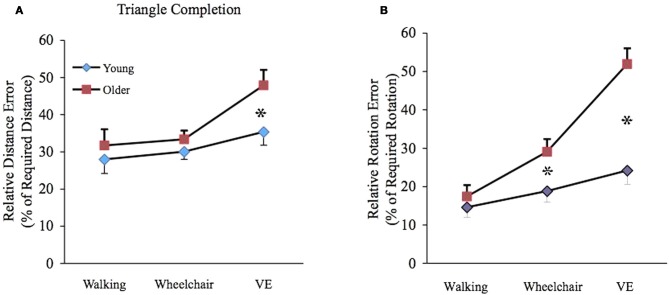
**(A)** Mean (± SE) relative distance error (RDE) for young (filled diamond) and older (filled square) participants. Age differences were significant in the VE condition (^*^p < 0.05). **(B)** Mean (± SE) relative rotation error (RRE) for young (filled diamond) and older (filled square) participants. Age differences were significant in the wheelchair (^*^p < 0.05) and VE (^*^p < 0.01) conditions.

Pairwise comparisons based on estimated marginal means determined that older individuals, mean (SE); 47.8 (4.1) generated greater relative distance errors (*p* < 0.05) than younger individuals mean (SE); 35.4 (3.6) in the VE condition. For rotation estimations, older individuals generated greater errors in the wheelchair (*p* < 0.05) and VE (*p* < 0.01) conditions mean (SE); 29.1 (3.2), 51.9 (4.1), respectively when compared to younger individuals mean (SE); 18.8 (2.8), 24.1 (3.5), respectively (see Figures 2A,B).

In young individuals, relative rotation error was greater in the VE 35.4 (3.6) than walking mean (SE); 27.9 (3.8) condition (*p* < 0.05) and in the wheelchair mean (SE); 30.6 (2.0) than walking condition, showing a tendency toward statistical significance (*p* = 0.06). There were no significant differences between conditions for relative distance error. However, for older individuals, relative distance error was greater in the VE mean (SE); 47.9 (4.1) than walking mean (SE); 31.7 (4.3) condition (*p* < 0.01) and in the VE mean (SE); 47.9 (4.1) than wheelchair mean (SE); 33.3 (2.3) condition (*p* < 0.01). Greater relative rotation error was found in the VE mean (SE); 51.9 (4.1) compared to the wheelchair mean (SE); 29.1 (3.2), condition (*p* < 0.01) and in the VE compared to the walking mean (SE); 17.4 (2.9) condition (*p* < 0.01).

Likewise, greater error was found in the wheelchair than walking condition (*p* < 0.01).

### Relative error for distance reproduction tasks

**Figure 3:**
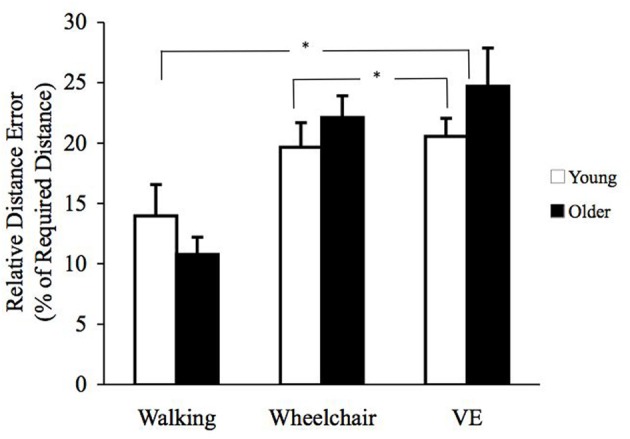
**Mean (± SE) relative distance error (RDE) for young (open) and older (filled) participants.** Conditions differences were found between VE and wheelchair; VE and walking conditions (^*^p's < 0.05).

A repeated measures ANOVA with age group as the between subjects factor and condition (walk, chair, and VE) and distance traveled (3.0, 6.1, and 9.1 m) as within-subject factors showed a main effect for condition [*F*_(2, 74)_ = 15.0, *p* < 0.01] and relative distance error as dependent variable. There were no significant main effects for age [*F*_(1, 36)_ = 0.28, *p* > 0.05] or distance traveled [*F*_(2, 72)_ = 0.1, *p* > 0.05]. Pairwise comparisons based on estimated marginal means showed that relative distance error was greater in the VE mean (SE); 22.6 (2.3) than wheelchair mean (SE); 20.8 (1.9) and walking mean (SE); 12.3 (2.0) conditions (*p*'s < 0.05) (see Figure 3).

## Discussion

Findings from this study showed that age differences in PI abilities were related to the availability of sensory information and task type (DRT, TCT). For DR tasks, older and younger individuals performed in a similar manner showing greater relative error in the VE than wheelchair condition, and in the wheelchair than walking condition. For TC tasks, older individuals were significantly further away from the origin than their younger counterparts for all conditions. When decomposing return to origin errors as a function of rotation and distance estimations, older individuals generated significantly greater rotation estimation errors in the wheelchair and VE conditions, and greater distance and rotation estimation errors in the VE condition when compared to young individuals.

### Triangle completion tasks

In the present study, there were larger errors when completing the TCT tasks in the VE than in real-world environments. This indicates that participants performed better at PI when they were blindfolded but allowed movement than when they were allowed vision but remained still. In support of this observation, previous work has found that proprioceptive/efferent sources of information have been shown to improve performance of a visually based VE task (Chance et al., 1998; Sun et al., 2004).

Indeed, locomotion was shown to enhance encoding of optic flow information used in VE TCT (Philbeck et al., 2001; Kearns et al., 2002) suggesting that the availability of vestibular and proprioceptive sensory information contributes to successful updating of movements (Loomis et al., 1993; Philbeck et al., 2008). In addition, Sun et al. (2004) showed that participants who remained stationary while viewing optic flow from a VE head mounted display performed less well in a TCT than subjects who walked or were transported in a wheelchair while viewing optic flow from a VE display. These findings suggest that optic flow by itself does not induce automatic updating of heading direction in a manner similar to that provided by vestibular and proprioception (Loomis et al., 2002). Our results are consistent with this interpretation.

When decomposing the relative contribution of rotation and distance estimations to TCT errors in the VE, greater errors were found for all rotation estimations across all triangles in older than young individuals. Similar errors in rotation were found for older individuals in other studies (Mahmood et al., 2009; Harris and Wolbers, 2012). Mahmood et al. (2009) showed that estimating the absolute rotation of a PI task contributed significantly to the overall error. However, Harris and Wolbers (2012) reported that rotation error was largely due to under rotating.

Further, the age effects found in the VE may be related to age-related differences in the processing of visual motion, especially optic flow (Atchley et al., 1998). Indeed, processing of self-motion cues is related to higher order processing (Wolbers et al., 2007) which may be vulnerable with aging. Kavcic et al. (2011) showed that older individuals required higher thresholds for detecting optic flow than younger individuals. In their study, participants were asked to judge which field on the visual display (left or right) was moving faster than the other. Older individuals required faster movements in opposing directions to distinguish differences in movement speed. However, this is in contrast to findings from others (Atchley et al., 1998; Billino et al., 2008a) who found optic flow detection to be unaffected by age suggesting that processing visual information is context-dependent and may be explained by differences in methodologies used to test for detection thresholds (Billino et al., 2008b). Indeed, Mossio et al. (2008) found estimating distances traveled in VE tasks when using optic flow were highly dependent on the strategy used by the participant and specific features associated with the presentation of visual information, such as texture regularity and depth cues, for example.

Allen et al. (2004) found no age difference in TCT in the walking condition. However, they found significant age differences in the same task following passive conveyance in the wheelchair, which they concluded, was largely the result of errors in estimating rotation. These findings are similar to the present results showing that the type of available sensory information distinguishes age differences in PI.

Tasks performed in the wheelchair condition restrict visual and proprioceptive feedback, forcing participants to rely on vestibular input. This finding is compatible with the observation that older individuals have greater difficulty using vestibular information when visual information is not available (Deshpande and Patla, 2007) and when there is decreased sensitivity of proprioceptors (Deshpande and Patla, 2007). In addition, as pointed out by Agrawal et al. (2009) degeneration of the vestibular system (Johnsson, 1971) may not be compensated for by proprioceptive information in older adults.

Vestibular and proprioceptive information also is critical for PI in animals (Mittelstaedt and Mittelstaedt, 1982; Etienne et al., 1988) Animals with vestibular damage showed declines in navigation when landmark cues were absent (Cohen, 2000; Stackman and Herbert, 2002), a finding which parallels the observation that labyrinthine-defective human subjects have difficulty estimating and reproducing the length of a path or a previously seen target (Glasauer et al., 1994, 1999).

### Distance reproduction tasks

Previous studies have shown that young individuals can accurately estimate path length following passive translation (Israel and Berthoz, 1989) and guided walking (Glasauer et al., 1994, 2002; Klatzky et al., 1990). However, no previous study has compared age differences in distance estimations across three different perceptual conditions.

In the present study, the DRT showed no age differences in performance, and both younger and older adults showed a similar magnitude of errors as a function of condition (VE>W/C>Walk). Our lack of age-differences in DRT is somewhat at odds with Mahmood et al. (2009) who showed that older individuals were less accurate in reproducing long (but not short) distances than younger individuals. However, there were substantial differences between studies in both the speed of movements and the distances traveled. In the Mahmood et al. (2009) study, distances ranged from 22.9 to 140.2 virtual m, and the speed of movement averaged 11.2 m/sec, which comes closer to replicating traveling by a motorized vehicle. Similarly, movement speeds were faster in the Harris and Wolbers (2012) study compared to the present study, which may explain a lack of age differences. In the present study, the VE speeds averaged 0.9 m/sec and distances traveled average 5.8 m. This suggests that when optic flow information is presented at slower speeds and/or over shorter distances, performance may improve in older adults resulting in negligible age differences for DRTs.

This investigation of age-related differences in PI abilities is limited by its relatively small sample size and cross sectional design, offering comparisons between older and young adults at a single time point. Indeed, longitudinal studies may offer a better representation of PI abilities when increasing age may be accompanied by declines in sensorimotor and cognitive function. In addition, the age range of the older adults (70.1 ± 6.4 years), health status and screening scores (MMSE scores > 28/30) indicate an active, healthy cohort, the results of which may not translate to a more at-risk population. Another potential limitation to the study was the lack of counterbalancing of task order. The VE condition was presented last to all participants leading to a possible confounding with subject fatigue that could potentially differ by age group. However, the fact that younger and older participants did not differ in fatigue at the end of the study suggests that this lack of counterbalancing likely played a minimal role in the age-differences observed.

We also acknowledge that factors other than sensory differences between conditions could contribute to the age differences observed herein. Among these additional factors may be age difference in working and episodic memory or spatial cognition and spatial imagery, age differences in interfacing with unfamiliar technological devices or possibly other factors that were not measured in the present study.

In sum, specific age and condition effects found in the present study may be explained with reference to the availability of sensory information (visual, proprioceptive, and vestibular). Findings from this study could be used to target interventions at reducing wayfinding difficulties in older adults. In addition, the paradigm used in this study is sensitive to detecting small changes in performance and future research may indicate its potential to serve as an indicator to track the onset and progression of dementia over time.

### Conflict of interest statement

The authors declare that the research was conducted in the absence of any commercial or financial relationships that could be construed as a potential conflict of interest.
